# Key biomarkers and latent pathways of dysferlinopathy: Bioinformatics analysis and *in vivo* validation

**DOI:** 10.3389/fneur.2022.998251

**Published:** 2022-09-20

**Authors:** Yan Xie, Ying-hui Li, Kai Chen, Chun-yan Zhu, Jia-ying Bai, Feng Xiao, Song Tan, Li Zeng

**Affiliations:** ^1^Department of Neurology, Sichuan Provincial People's Hospital, University of Electronic Science and Technology of China, Chengdu, China; ^2^Chinese Academy of Sciences Sichuan Translational Medicine Research Hospital, Chengdu, China; ^3^Department of Neurology, People's Hospital of Yilong County, Nanchong, China

**Keywords:** dysferlinopathy, bioinformatics analysis, biomarker, immune cell infiltration, ubiquitin-proteasome pathway

## Abstract

**Background:**

Dysferlinopathy refers to a group of muscle diseases with progressive muscle weakness and atrophy caused by pathogenic mutations of the *DYSF* gene. The pathogenesis remains unknown, and currently no specific treatment is available to alter the disease progression. This research aims to investigate important biomarkers and their latent biological pathways participating in dysferlinopathy and reveal the association with immune cell infiltration.

**Methods:**

GSE3307 and GSE109178 were obtained from the Gene Expression Omnibus (GEO) database. Based on weighted gene co-expression network analysis (WGCNA) and differential expression analysis, coupled with least absolute shrinkage and selection operator (LASSO), the key genes for dysferlinopathy were identified. Functional enrichment analysis Gene Ontology (GO) and Kyoto Encyclopedia of Genes and Genomes (KEGG) were applied to disclose the hidden biological pathways. Following that, the key genes were approved for diagnostic accuracy of dysferlinopathy based on another dataset GSE109178, and quantitative real-time polymerase chain reaction (qRT-PCR) were executed to confirm their expression. Furthermore, the 28 immune cell abundance patterns in dysferlinopathy were determined with single-sample GSEA (ssGSEA).

**Results:**

1,579 differentially expressed genes (DEGs) were screened out. Based on WGCNA, three co-expression modules were obtained, with the MEskyblue module most strongly correlated with dysferlinopathy. 44 intersecting genes were recognized from the DEGs and the MEskyblue module. The six key genes *MVP, GRN, ERP29, RNF128, NFYB* and *KPNA3* were discovered through LASSO analysis and experimentally verified later. In a receiver operating characteristic analysis (ROC) curve, the six hub genes were shown to be highly valuable for diagnostic purposes. Furthermore, functional enrichment analysis highlighted that these genes were enriched mainly along the ubiquitin-proteasome pathway (UPP). Ultimately, ssGSEA showed a significant immune-cell infiltrative microenvironment in dysferlinopathy patients, especially T cell, macrophage, and activated dendritic cell (DC).

**Conclusion:**

Six key genes are identified in dysferlinopathy with a bioinformatic approach used for the first time. The key genes are believed to be involved in protein degradation pathways and the activation of muscular inflammation. And several immune cells, such as T cell, macrophage and DC, are considered to be implicated in the progression of dysferlinopathy.

## Introduction

Dysferlinopathy, caused by mutation in the *DYSF* gene, is a family of autosomal recessive muscular disorders with distinguishing clinical features. Although with a low overall incidence, dysferlinopathy accounts for as much as 30% of progressive recessive muscular dystrophies in some geographic areas (1). Most commonly the symptoms present in late adolescence with progressive muscle weakness, and the most common phenotypes are limb girdle muscular dystrophy R2 (LGMD R2) and Miyoshi distal myopathy depending on primary involvement of proximal or distal muscles (2). Currently, dysferlinopathy does not have any effective treatment, and the molecular mechanism determining its pathogenesis remains unknown.

Dysferlin is a 237 kDa transmembrane protein that plays a critical role in membrane repair and vesicle trafficking (3). Patients with dysferlinopathy typically have dysferlin labeling loss in muscle biopsies. Evidence from clinical and experimental studies suggests that inflammatory- and immune-related pathways contribute to dysferlinopathy progression. In dysferlinopathy, necrosis, regeneration, inflammation and upregulation of major histocompatibility complex class I (MHC-I) are the most common pathological features, causing the condition to be misdiagnosed as inflammatory myopathy (4). In contrast to polymyositis, Duchenne muscular dystrophy or Becker muscular dystrophy (DMD/BMD), CD4 T cell, macrophage, and membrane attack complex (MAC) are more highly expressed in muscles with dysferlinopathy (5). Researchers have shown that oxidative stress activates NF-κB p65 signaling and may contributes to muscle protein loss in dysferlinopathy (6). However, the treatment with prednisolone or the inhibition of inflammation with celastrol fails to improve muscle function in mice with dysferlin deficiency (7, 8). It is largely unknown how the mutation of the single gene *DYSF* could induce muscular inflammation, and there is no effective treatment available for this condition. In order to better understand the pathophysiologic mechanisms behind muscle pathology and provide potential treatments, hub genes and key pathways associated with dysferlinopathy must be investigated.

Recent advancements in microarray technologies and bioinformatics have made it possible to investigate disease pathogenesis and discover biomarkers for disease progression and therapeutic efficacy. In this study, we used WGCNA in combination with LASSO to identify the key biomarkers of dysferlinopathy, and then functional enrichment analysis to explore cellular pathways. The key genes were also validated *in vivo*. Ultimately, we analyzed the infiltration of 28 immune cells in dysferlinopathy with single-sample GSEA (ssGSEA). As far as we know, this is the first to examine immune cell infiltration and key gene expression in dysferlinopathy, revealing a more complete picture of its pathogenesis.

## Materials and methods

### Datasets of dysferlinopathy

**Figure 1:**
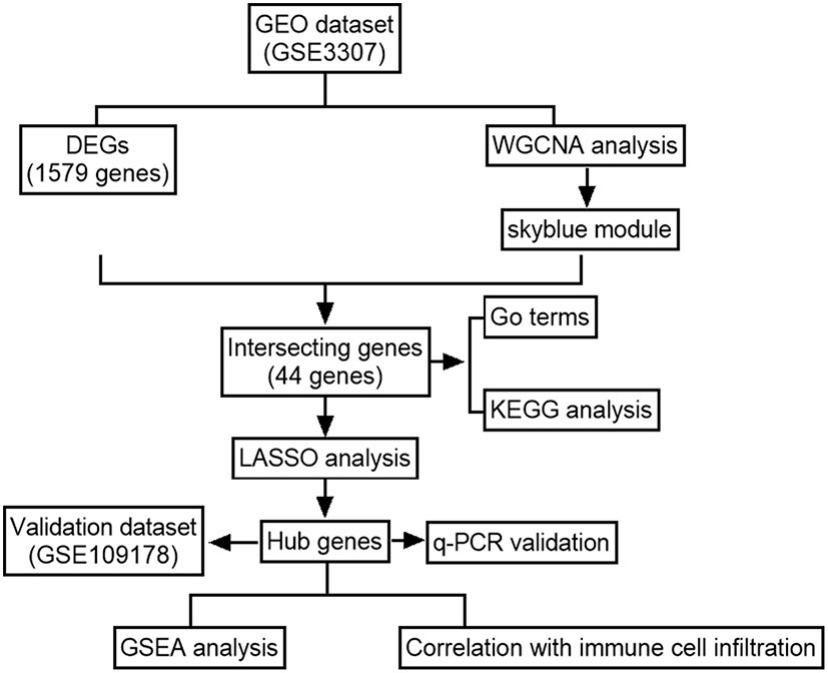
Flowchart of the analysis.

Figure 1 illustrates the workflow for bioinformatics analysis. The dysferlinopathy RNA-sequencing datasets GSE3307 and GSE109178 were obtained from the GEO databank (Gene Expression Omnibus, https://www.ncbi.nlm.nih.gov/geo/). GSE3307 contains 10 dysferlinopathy samples and 18 normal samples, while GSE109178 contains 8 dysferlinopathy samples and 6 normal samples. Using the R package “affy,” the original data were analyzed and interpreted.

### WGCNA analysis

The GSE3307 dataset was used to construct a signed co-expression network with the WGCNA package in R (9). In detail, a variance calculation was conducted for each gene expression value, and a subset of genes with absolute deviations of more than 25% from the median was extracted for analysis (10). The “goodSampleGenes” function was applied to include qualified samples, then the “pickSoftThreshold” function was used to determine an ideal soft threshold (β) to construct the weighted adjacency matrix. Next, the adjacency matrix was converted into a topological matrix (TOM), and the genes were grouped by using the flashClust function. The genes were grouped into modules as per expression pattern similarity using a hybrid dynamic tree cutting algorithm, with a minimum of 30 genes in each module. A MEDissThres (the dissimilarity threshold of module eigengenes) = 0.2 was used to consolidate the similar modules to yield the ultimate modules. The association between modules and the phenotype of interest was revealed by Pearson correlation analysis. The module with the highest correlation with the phenotype of interest were chosen as the module of interest. To determine gene significance (GS) and module membership (MM), the modules and phenotypic data were input into the co-expression network. MM is the relationship between genes and modules; GS is the linkage of gene expression and phenotypes, which is calculated by log10 transformation of the *P*-value (GS = lgP) in the linear regression (11). Each candidate gene with |GS|>0.2 and |MM|>0.8 was screened out to categorize as a dysferlinopathy-related module gene.

### Differential genes expression analysis

A gene expression abnormality analysis of dysferlinopathy and control samples in the GSE3307 dataset was implemented with the “LIMMA” package in R software. Log_2_|fold change (FC)|>0.7 adjusted *P*-value<0.05 were the screening criteria.

### Key gene screening and validation

Common elements between dysferlinopathy-related module genes and differentially expressed genes (DEGs) were revealed using the Jvenn online tool-based cross-tabulation function in http://jvenn.toulouse.inra.fr/app/example.html. A LASSO analysis was then performed using the new glmnet package of R software to screen for the key genes (12). The GraphPad Prism software (version 9.3) was used to calculate expression levels of the six genes in dysferlinopathy and controls. The receiver operating characteristic (ROC) curves were calculated with the “ROCR” package to assess the ability of key genes to distinguish dysferlinopathy from controls. Furthermore, the expression levels and diagnostic value of key genes were validated by using a separate external dataset GSE109178.

### GO and KEGG enrichment analysis

In R, the “enrichplot” and “clusterProfiler” packages were used for functional annotations, including Gene Ontology (GO) and Kyoto Encyclopedia of Genes and Genomes (KEGG). An enrichment of terms/pathways matching *p* < 0.05 was recognized as statistically significant. The top ten terms were visualized using the “ggplot2” package.

### Gene set enrichment analysis (GSEA)

In order to elucidate their hidden functions of the key genes, the samples were grouped into high and low expression series according to their median expression values. The GSEA software (version 4.4.3) from the Broad Institute (http://software.broadinstitute.org/gsea/downloads.jsp) was used to analyze KEGG enrichment in the high and low expression segments (13). The C2 curated gene set database was freely available from the Molecular Signature Database (MsigDB) as a reference for the KEGG analysis. The gene set arrangement was performed 1,000 times per analysis. Those with |NES|≥1 and *P*-value<0.05 were regarded as significantly enriched pathways.

### Assessment of immune cell abundance in dysferlinopathy

In dysferlinopathy tissues and controls, 28 immune cells were quantified using single-sample gene set enrichment analysis (ssGSEA). A boxplot was drawn to show the differential expression of the 28 immune infiltrating cells between the two groups. With the “ggplot2” package, Spearman correlations were analyzed between the key genes and the 28 immune infiltrating cells.

### RNA extraction and quantitative real-time polymerase chain reaction (qRT-PCR)

Four patients with dysferlinopathy were diagnosed based on their clinical features, muscle biopsy, dysferlin immunostaining and *DYSF* gene sequencing. For non-dysferlinopathy control, the muscle samples were collected from 4 patients without a muscle disease as per clinical, histologic, and EMG criteria. Supplementary Table S1 provides relevant clinical information. Muscle samples were obtained and stored at −80°C for further experiment. The RNA from muscles was prepared with Trizol reagent (Thermo Fisher Scientific, Waltham, USA) in accordance with the manufacturer's protocol. The extracted RNA was reverse-transcribed to cDNA using PrimeScript RT reagent kit (Takara, Japan). Quantitative PCR was performed with the TB Green Premix Ex Taq II (Takara, Japan) following the manufacturer's instructions. PCR amplification was performed for 30 s at 95°C and followed by 40 cycles of 5 s at 95°C 30 s at 55°C, and 30 s at 72°C. The 2^−ΔΔCT^ method was used to determine the relative expression of the s key mRNAs. The primers are listed in Supplementary Table S2.

### Statistical analysis

Statistics were conducted using GraphPad Prism 9.3. A Student's non-parametric *t*-test (Mann-Whitney test) with a *P*-value of 0.05 was used to compare the two groups.

## Results

### Identification of dysferlinopathy-related module genes

**Figure 2:**
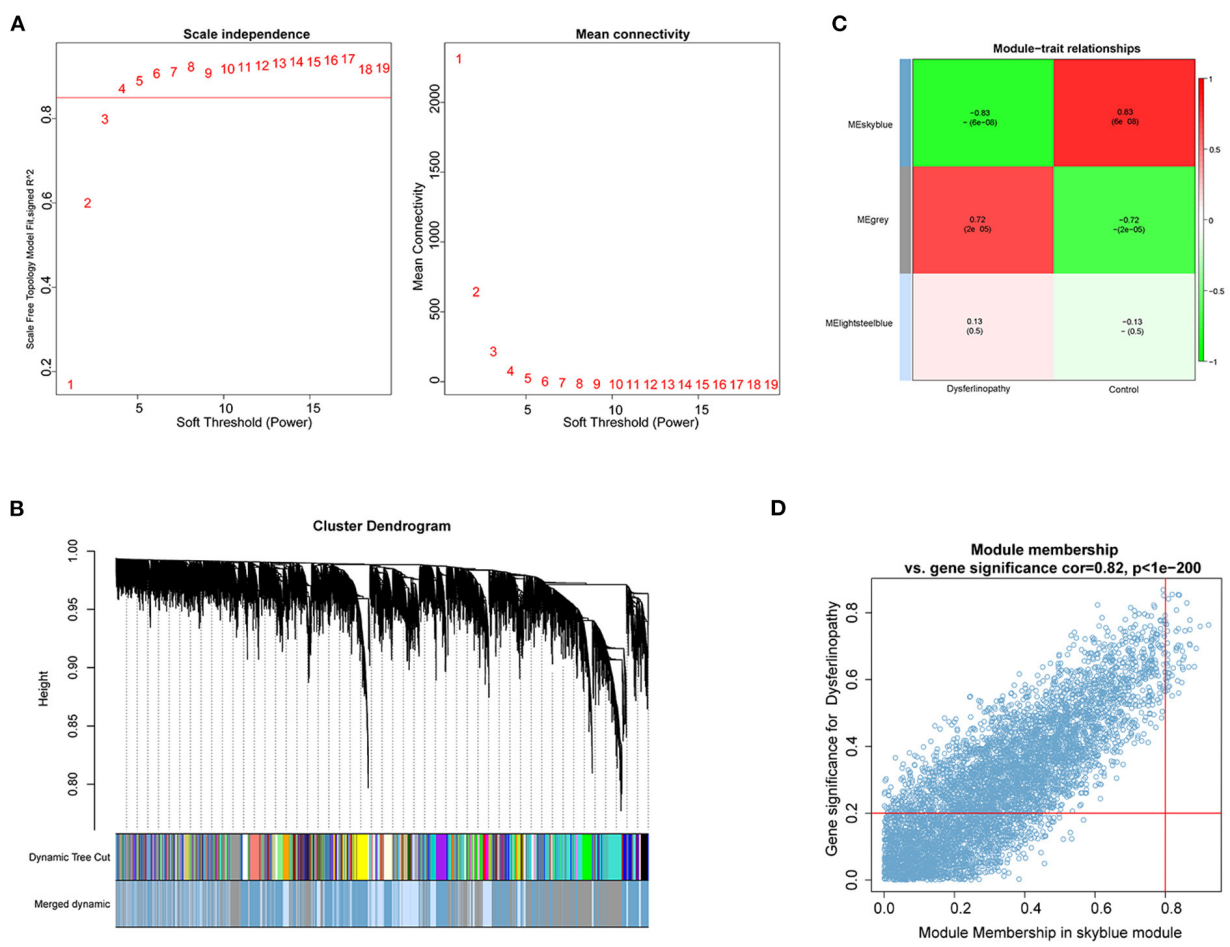
Identification of key module based on WGCNA analysis. **(A)** Calculation of soft-thresholding power. A correlation coefficient of 0.9 and a soft-thresholding power of four are shown on the red line. **(B)** Gene cluster dendrogram on the basis of module eigengenes. The different colors represent different modules. **(C)** Module gene and clinical phenotype correlation analysis. The MEskyblue module was significantly associated with dysferlinopathy. **(D)** Scatter plot for correlation between genes within the MEskyblue module and clinical phenotype data.

With the R package WGCNA, we implemented co-expression network analysis on the GSE3307 dataset. The expression information from the dataset was used as input, and the total samples were analyzed by hierarchical clustering. Outlier samples (height >150) were excluded from subsequent analyses in WGCNA. No samples were eliminated due to high heterogeneity (Supplementary Figure S1). A soft threshold of β = 4 was chosen for the scale-free network, and then the co-expression matrix was conducted (Figure 2A). After module merging, three gene modules were obtained (Figure 2B). Based on the Pearson correlation heat maps of the interest phenotypes (dysferlinopathy) and modules, the most attractive module was the MEskyblue module (cor = 0.83; *p* = 6e−8) as shown in Figure 2C. Furthermore, analyses of the genes in the MEskyblue module and dysferlinopathy clinical phenotypes showed a strong correlation (cor = 0.82; *p* < 1e−200) (Figure 2D). Therefore, the MEskyblue module was deemed as the dysferlinopathy-related module. A total of 61 genes were defined as module genes affiliated with dysferlinopathy for subsequent analysis based on |GS| >0.2 and |MM| >0.8.

### Recognition of DEGs in dysferlinopathy and screening of key genes

**Figure 3:**
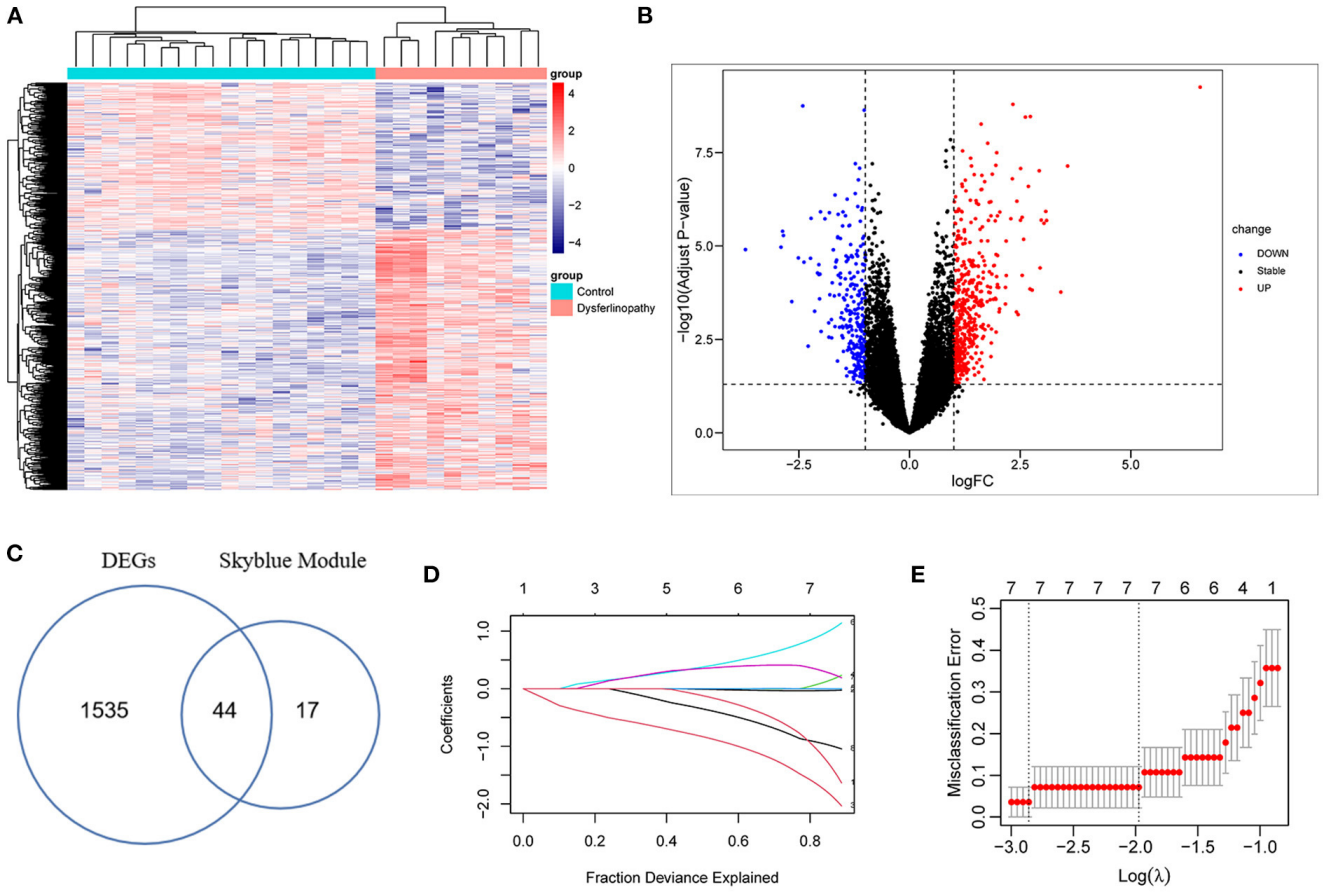
Analysis of DEGs in GSE3307 dataset and screening of key genes. **(A)** Heat map of the DEGs in dysferlinopathy. **(B)** Volcano plot showing DEGs between dysferlinopathy muscles and normal controls. **(C)** Venn diagram for intersections between MEskyblue module and DEGs. **(D)** The partial likelihood deviance curve was plotted vs. log (λ) in 10-fold cross-validations. **(E)** Seven key genes screened by LASSO regression analysis in a cross-validation of 10 fold.

After normalization of the GSE3307 dataset (Supplementary Figure S2), 1579 DEGs (683 up-regulated and 896 down-regulated genes) were extracted based on the defined criteria. The DEGs are visualized in the heat map (Figure 3A) and volcano plot (Figure 3B). Furthermore, 44 common genes were derived from the intersection of 61 dysferlinopathy-related module genes and 1,579 DEGs (Figure 3C). The LASSO algorithm was then executed, and the following seven key genes were identified: *MVP, GRN, ERP29, RNF128, NFYB, KPNA3* and *PRKN* (Figures 3D,E).

### GO and KEGG enrichment analysis of intersecting genes

**Figure 4:**
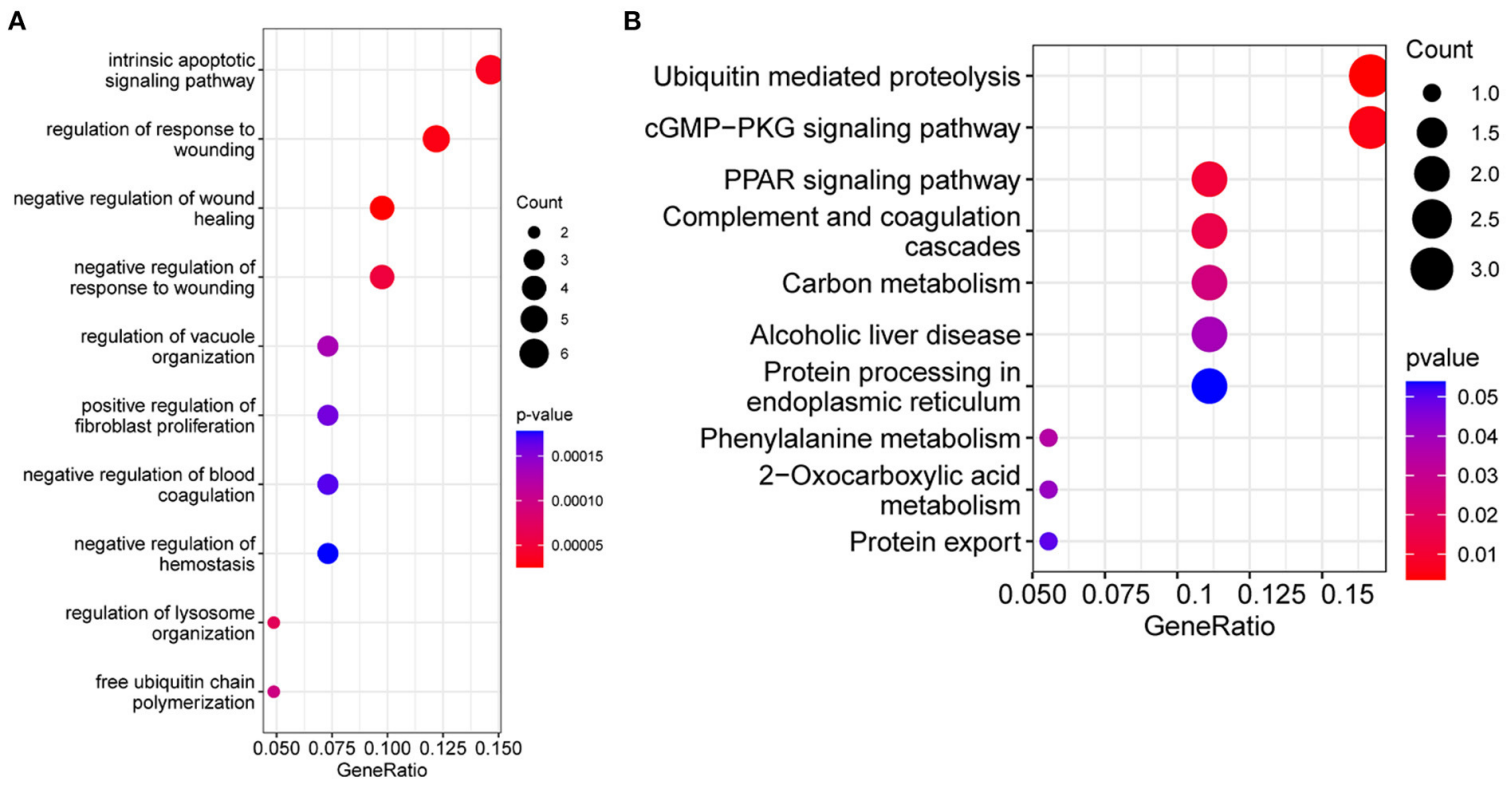
Functional enrichment analysis results of the intersecting genes between the MEskyblue module and the DEGs. **(A)** Bubble diagram of the top 10 GO terms. **(B)** The top 10 KEGG pathways. The size of bubbles indicates number of enrichment genes, and the color indicates value.

In order to understand the biological pathways associated with dysferlinopathy, 44 intersecting genes were analyzed using enriched GO and KEGG pathways. In terms of the GO, these genes were mainly relevant with intrinsic apoptotic signaling pathways, wounding and wound healing process, and protein degradation pathways (e.g., regulation of lysosome organization and free ubiquitin chain polymerization) (Figure 4A). In terms of the KEGG functional enrichment analysis, the gene set was significantly enriched in ubiquitin-mediated proteolysis, cGMP-PKG signaling pathway, peroxisome proliferator-activated receptors (PPAR) pathway, protein processing in endoplasmic reticulum (ER) and so forth (Figure 4B).

### Evaluation of key gene expression levels and diagnostic value

**Figure 5:**
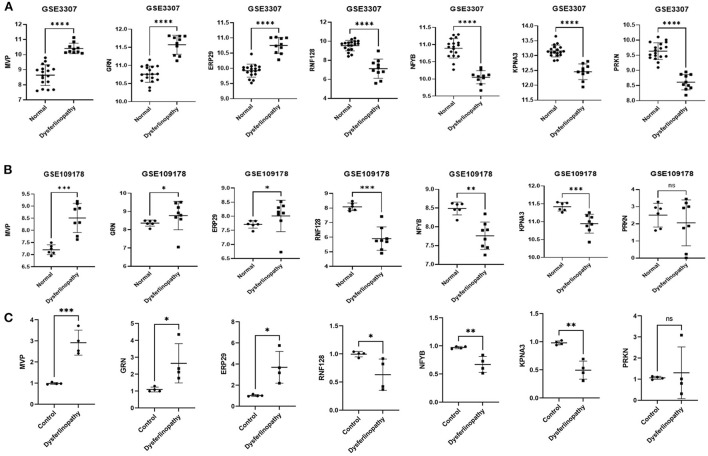
Expression validation of seven genes. **(A)** Expression of *MVP, GRN, ERP29, RNF128, NFYB, KPNA3* and *PRKN* in dysferlinopathy (*n* = 10) compared with controls (*n* = 18) in the GSE3307 dataset. **(B)** Expression of *MVP, GRN, ERP29, RNF128, NFYB, KPNA3* and *PRKN* in dysferlinopathy (*n* = 8) compared with controls (*n* = 6) in the GSE109178 dataset. *PRKN* was the only gene with an expression level not statistically significant in the GSE109178 dataset. **(C)** Expression of *MVP, GRN, ERP29, RNF128, NFYB, KPNA3* and *PRKN* in four dysferlinopathy patients compared to four controls by qRT-PCR. A Student's non-parametric *t*-test with a *P*-value of 0.05 was taken to compare the two groups. *p<0.05; **p<0.01; ***p<0.001; ****p<0.0001; ns, non significance.

**Figure 6:**
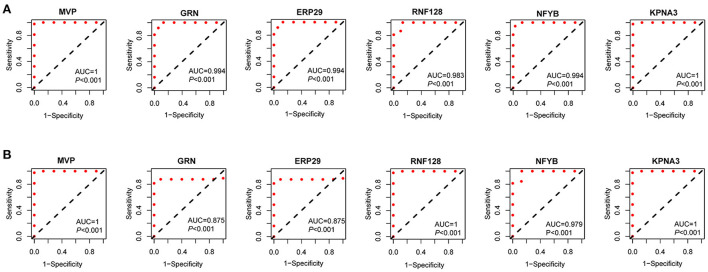
Diagnostic efficiency of the seven key genes. **(A)** The ROC curves of the key genes in the GSE3307. **(B)** The ROC curves of the key genes in the GSE109178.

The expression levels of *MVP, GRN, ERP29, RNF128, NFYB, KPNA3* and *PRKN* in GSE3307 and GSE109178 were compared. In both datasets, *MVP, GRN*, and *ERP29* showed significantly higher expression levels, while *RNF128, NFYB*, and *KPNA3* had significantly lower expression levels in dysferlinopathy than normal controls (Figures 5A,B). The only gene with an expression level showing no statistically significant difference between dysferlinopathy tissues and controls in GSE109178 dataset was *PRKN*, so this gene was excluded from the key gene list. To further validate the identified key genes *in vivo*, muscle tissues were extracted from dysferlinopathy patients and controls to confirm whether the mRNA levels of the key genes in these samples were consistent with the bioinformatics analysis. Consistent with the two datasets, *MVP, GRN*, and *ERP29* expression were significantly upregulated, and *RNF128, NFYB*, and *KPNA3* were significantly downregulated in the dysferlinopathy patients compared with the controls (Figure 5C). Furthermore, the six genes were validated in other muscular disorders, including Limb-girdle muscular dystrophy 2A (LGMD2A), facioscapulohumeral muscular dystrophy (FSHD), dermatomyositis and polymyositis. As with dysferlinopathy, the six genes were significantly dysregulated in LGMD2A tissues compared to controls (Supplementary Figure S3A). Intriguingly, only *MVP* was statistically significant differences between FSHD muscles and healthy controls (Supplementary Figure S3B). In polymyostis and dermatomyositis, *MVP* and *GRN* expression levels were significantly higher, while *NFYB* levels were significantly lower (Supplementary Figures S3C,D). To evaluate the diagnostic value of the six key genes, the area under the curve (AUC) values were determined. The AUC values of all six key genes in the GSE3307 dataset were > 0.95, showing their high diagnostic value for dysferlinopathy (Figure 6A). Furthermore, all six key genes had AUC values above 0.85 in the GSE109178 dataset, supporting their diagnostic value in dysferlinopathy (Figure 6B). In the LGMD2A and FSHD datasets, all six genes were diagnostically significant for LGMD2A, but *MVP* was the only diagnostically significant gene for FSHD with an AUC value of 0.808 (Supplementary Figures S4A,B). *NFYB, GRN* and *MVP* were diagnostically significant for both dermatomyositis and polymyositis in the dataset for inflammatory myopathy (Supplementary Figures S4C,D).

### Genomic enrichment analysis

**Figure 7:**
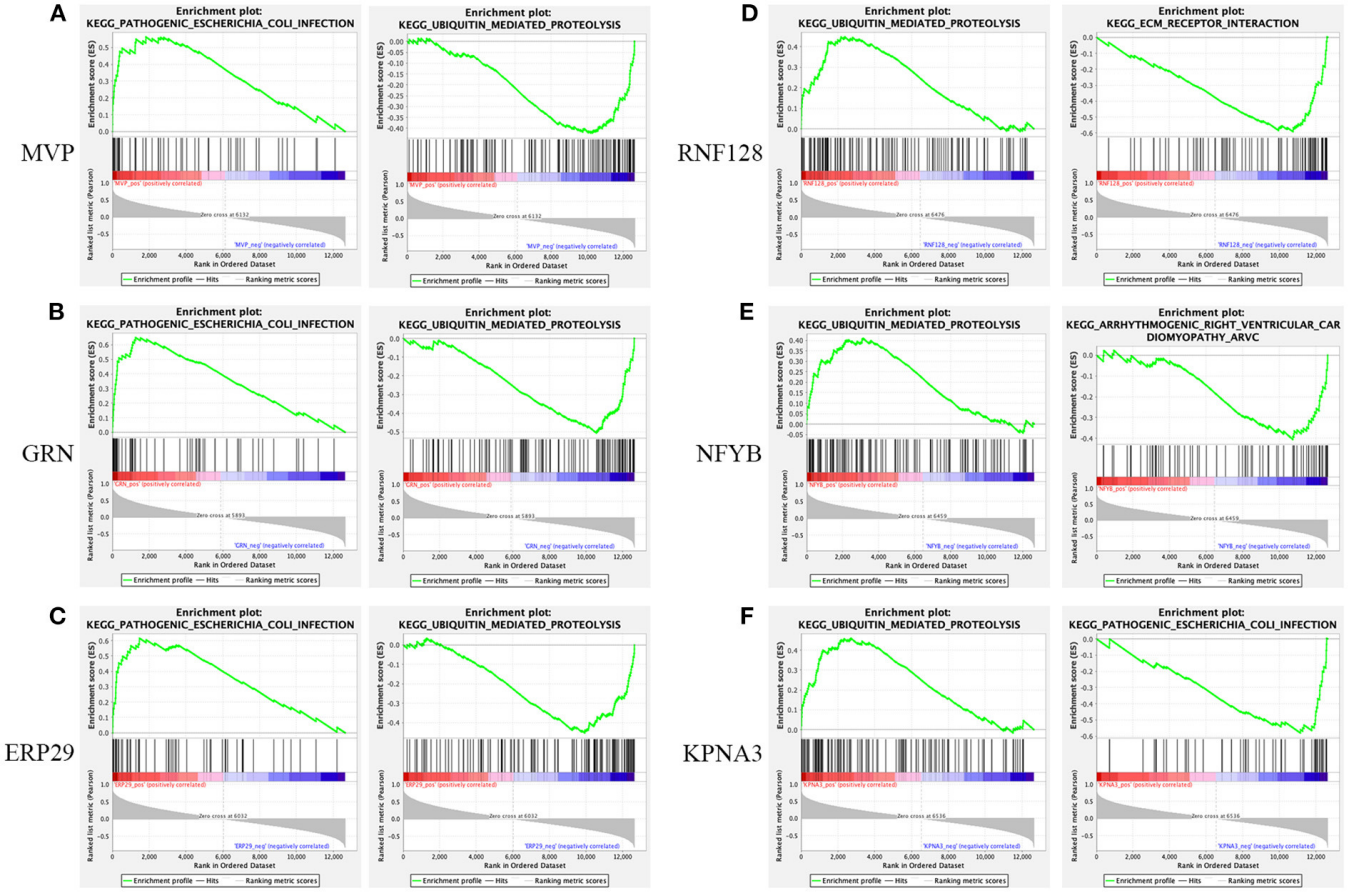
Gene set enrichment analysis. KEGG entries most positively and negatively linked to *MVP* **(A)**, *GRN* **(B)**, *ERP29* **(C)**, *RNF128* **(D)**, *NFYB* **(E)**, and *KPNA3* **(F)**.

Using native GSEA software, we administered gene set enrichment analysis for each of the six key genes individually to investigate their potential biological functions in dysferlinopathy progression. Figure 7 had showed the KEGG pathways most positively and negatively linked to the six genes. It revealed that all the six genes were engaged in the ubiquitin-mediated proteolysis pathway, and most of the genes were enriched in pathogenic E-coli infection (Figures 7A–F).

### Immune landscape in dysferlinopathy and its correlation with key genes

**Figure 8:**
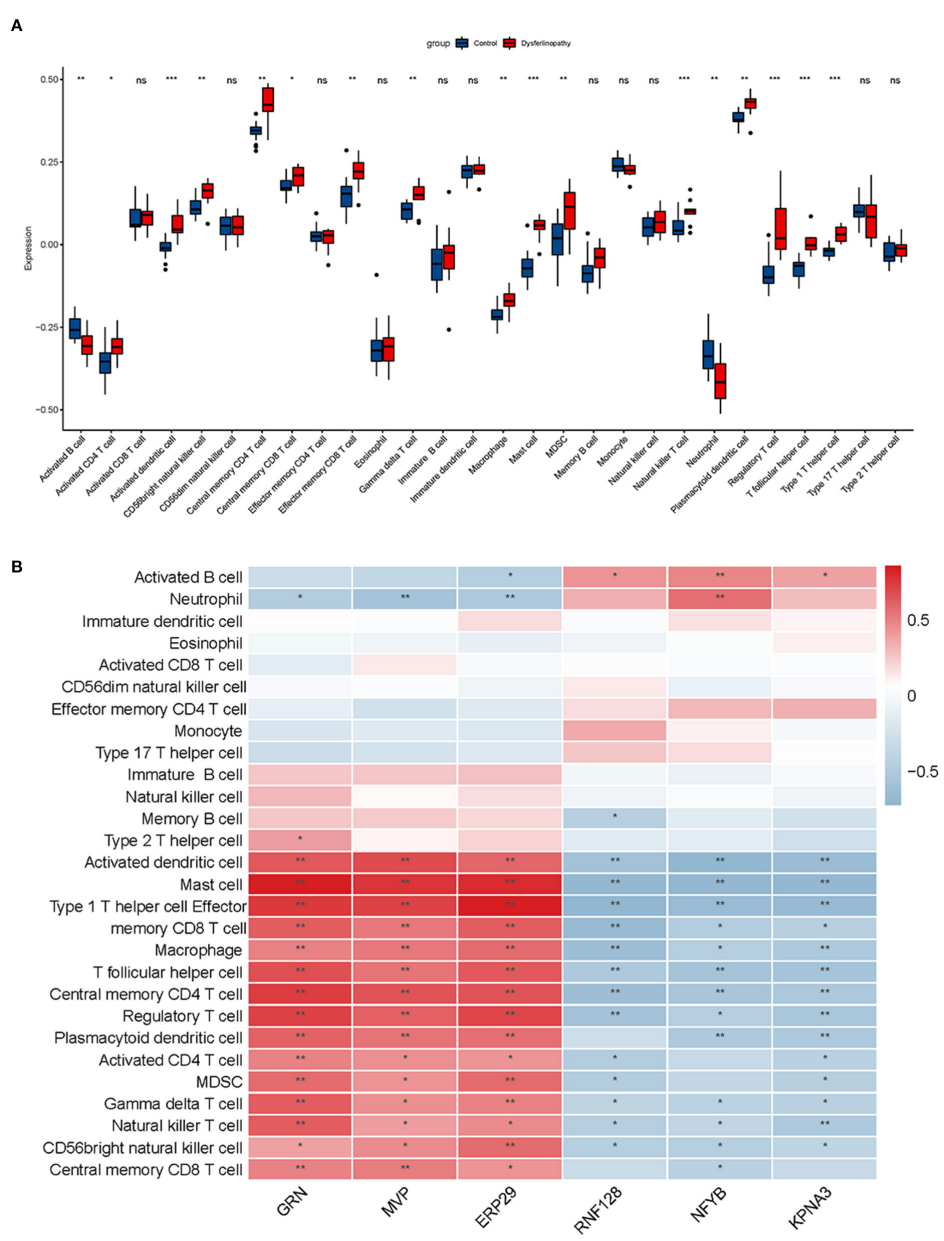
Immune infiltration analysis associated with dysferlinopathy. **(A)** A comparison of 28 types of immune cells between dysferlinopathy and controls. **(B)** Infiltration levels of immune cells and six key genes in a heat map. Red indicates a strong positive correlation, whereas blue indicates a strong negative correlation. *p< 0.05; **p< 0.01.

Dysferlinopathy, which mimics polymyositis, shows necrosis, regeneration and inflammatory cell infiltration along with high MHC-I expression in muscle biopsy. Therefore, we assessed the degree of immune cell infiltration in dysferlinopathy compared with controls using ssGSEA. As shown in Figure 8A, 17 out of 28 immune cells illustrated higher infiltration in the dysferlinopathy muscles, such as CD4 T cells, CD8 T cells, natural killer T cells, regulatory T cells, type I T helper cells, macrophage, activated dendritic cells (DC), neutrophils. Analyzing of the 28 immune cells with six key genes, the abundance of immune cell infiltration was positively correlated with *GRN, MVP, ERP29*, but negatively correlated with *RNF128, NFYB, KPNA3* (Figure 8B).

## Discussion

Dysferlinopathy is a group of autosomal recessive inherited myopathies caused by mutations in the *DYSF* gene. Mutations in the *DYSF* gene may cause either LGMD2B or a Miyoshi myopathy phenotype. The exact pathogenesis of dysferlinopathy is unclear, and no treatment is available yet. In this study, using bioinformatics analysis, we identified the key genes and important pathways associated with the disease, and uncovered the immune infiltration microenvironment in dysferlinopathy.

Microarray technology application has allowed bioinformatics researchers to identify key genes involved in disease pathogenesis in an efficient and rapid manner, providing a new avenue for diagnosis and treatment of diseases. In this study, using GO and KEGG functional enrichment analyses, it was found that the identified DEGs were largely involved in ubiquitin-proteasome pathway (UPP),would healing, PPAR signaling pathway and protein processing in ER. GSEA functional enrichment results demonstrated all the six key genes were involved in the ubiquitin-mediated proteolysis pathway. These terms provide insight into the molecular mechanisms of dysferlinopathy and may provide treatment targets for the disease. In dysferlinopathy, UPP is important (14, 15). Dysferlin depletion activates the inflammasome pathway, which increases monocyte phagocytosis (16, 17). Mutant dysferlin has been found to aggregate on the ER, and can be degraded by the UPP and autophagy-lysosome system (18). Muscle-specific ubiquitin E3 ligase RING-finger protein-1 (MuRF1) is found elevated in both mRNA and protein levels, and the overall amount of ubiquitinated muscle proteins is higher in dysferlinopathy (6, 19). Conversely, inhibition of proteasome activation results in a trend toward increased dysferlin and alleviates muscle inflammation (20), suggesting an important role of UPP in dysferlinopathy. The best agreement among these studies and the enrichment results indicate that the UPP plays a key role in dysferlinopathy onset and progression, and may act as a therapeutic target.

Using WGCNA and LASSO algorithms and *in vivo* experiments, we have identified six key genes: *MVP, GRN, ERP29, RNF128, NFYB* and *KPNA3*. Significant abnormality in the expression levels of the six genes were revealed in dysferlinopathy, with *MVP, GRN* and *ERP29* genes significantly upregulated, while *RNF128, NFYB* and *KPNA3* significantly downregulated. Endoplasmic reticulum protein 29 (ERP29) is localized in the ER lumen and serves as a chaperone that facilitates the transport of proteins from the ER to the Golgi apparatus (21). It plays a key role in promoting protein degradation and preventing protein aggregation by removing misfolded proteins from the ER. Under stress conditions, ERP29 upregulates chaperones involved in stress response pathways and promotes cell survival (22). When unfolded/misfolded proteins are not eliminated and accumulate in the ER system, they cause ER homeostasis to be out of balance, which promotes apoptosis and inflammation in cells (14). There is a strong possibility that *ERP29* is upregulated in dysferlinopathy as a compensatory mechanism. The ring finger protein 128 (RNF128) is a type I transmembrane protein containing a ring zinc-finger motif and is an E3 ubiquitin ligase. By catalyzing the formation of polyubiquitin chains linked to lysine 48 and lysine 63, it inhibits the transcription of cytokine genes. Increasing RNF128 expression in T cells negatively affects IL-2 and IL-4 production, and induces anergic phenotypes (23). Significantly low expression of RNF128 in dysferlinopathy may lead to increased production of cytokines, and cause muscle inflammation. Progranulin is encoded by the Granulin gene (GRN) and is primarily present in the lysosome membrane. As a key regulator of lysosome function, progranulin allows for protein trafficking and lysosome acidification, and may also affect wound healing, inflammation, and cell proliferation (24, 25). A decrease in *GRN* expression induces neutrophil migration and cytokine response in neurons. Numerous studies have shown that down-regulation of *GRN* expression is associated with inflammation in multiple neurodegenerative diseases such as amyotrophic lateral sclerosis, frontotemporal dementia, Alzheimer's disease and Parkinson's disease (26). However, *GRN* expression is up-regulated in dysferlinopathy, which strongly suggests that the lysosome pathway is over-expressed, perhaps as a protective compensation mechanism. Karyopherin alphas (KPNAs) transport proteins into and out of the nucleus through the nuclear pore complex (27). In Drosophila, KPNA3 is involved in transporting heat shock transcription factors into the nucleus. When KPNA3 is knocked down, heat shock protein does not enter the nucleus, causing heat shock response to end (28). A downregulation of *KPNA3* expression in dysferlinopathy may exacerbate the disease by terminating the heat shock response. The major vault protein (MVP) is a multi-subunit ribonucleoprotein structure that may participate in nucleocytoplasmic transport. In a recent review, Berger *et al*. explored the effects of MVP in multiple intracellular transduction as well as immune defense and inflammation caused by infectious disease (29). The protein MVP is largely elevated in rheumatoid arthritis and other inflammatory diseases (30). In the above mentioned studies, *ERP29, RNF128, GRN* and *KPNA3* are closely related to protein-degradation pathways and protein processing in ER, while *MVP* is associated with inflammation. Dysregulation of these genes impairs misfolded protein degradation, induces ER stress and muscle inflammation, and maybe contribute to the progression of dysferlinopathy. As inflammation is a secondary pathological change, anti-inflammatory therapies with prednisone or celasterol cannot effectively address the disease cause. Currently, it is unclear if NFYB contributes to dysferlinopathy progression and more studies are needed. Additionally, we examined the expression levels and diagnostic values of the key genes involved in hereditary and inflammatory myopathy. In LGMD2A tissues, the six key genes had similar diagnostic significance to dysferlinopathy, but only *MVP* was diagnostically significant in FSHD. There appears to be an association between LGMD2A and dysferlinopathy, which shares common pathogenic molecular mechanisms that are strikingly different from those of FSHD. In inflammatory myopathies, *GRN, MVP* and *NFYB* showed high diagnostic value, while *RNF128, ERP29* and *KPNA3* showed poor diagnostic value, indicating high inflammation activation but little involvement of protein homeostasis.

Based on the above functional enrichment analysis and the key genes, their biological function was mainly enriched in protein degradation pathway, and dysregulation of these genes resulted in inflammation activation. Due to the fact that immune cell infiltration is a common pathogenic feature in dysferlinopathy, we analyzed the immune infiltration landscape in dysferlinopathy using the ssGSEA algorithm for the first time. Compared to normal controls, dysferlinopathy muscles had significantly more T cells (including CD4 T cell, CD8 T cell, natural killer T cell, regulatory T cell, type I T helper cell), macrophages, and dendritic cells. The key genes also significantly affected the infiltration of multiple immune cells. In agreement with our results, previous studies have shown that CD4 cells, macrophages, MHC-I and C5b-9 are positive in immunohistochemically stained dysferlinopathy muscles (5). Among dysferlin-deficient mice, Urao *et al*. reported that macrophage activity was significantly correlated with disease progression (31, 32). When B and T lymphocytes were removed from dysferlinopathy animal models, muscle regeneration was improved (33). These findings provided preliminary insight into dysferlinopathy's immune infiltration pattern, and confirmed that disturbances in immune homeostasis are crucial to dysferlinopathy progression.

However, our study has several limitations. First, the dataset for analysis was obtained using old microarray technology rather than RNA sequencing. Secondly, we simply verified the results of bioinformatics analysis in experimental studies, further studies of these key genes and related pathways must be conducted to confirm our findings.

In summary, based on WGCNA and LASSO, combined with ssGSEA, six key genes (*MVP, GRN, ERP29, RNF128, NFYB*, and *KPNA3*) involved in dysferlinopathy have been identified, and their most important functions relate to protein degradation pathways, which in turn influence immune cell infiltration in dysferlinopathy. In a sense, the key genes are considered as potential biomarkers or drug targets for dysferlinopathy.

## Data availability statement

Publicly available datasets were analyzed in this study. This data can be found here: National Center for Biotechnology (NCBI) Gene Expression Omnibus (GEO), http://www.ncbi.nlm.nih.gov/ (GSE3307 and GSE109178).

## Ethics statement

The studies involving human participants were reviewed and approved by Ethics Committee of the Sichuan Provincial People's Hospital. The patients/participants provided their written informed consent to participate in this study.

## Author contributions

YX, ST, and LZ designed the research. C-yZ, KC, FX, and J-yB gathered clinical information as well as muscle samples of dysferlinopathy patients and controls. Y-hL performed the experimental work and drafted the manuscript. YX and LZ performed bioinformatic analysis. All authors read and revised the final manuscript before submission.

## Funding

This work was supported by the National Natural Science Foundation of China (Code. 81801270) and by the Sichuan Provincial People's hospital (Code. 2017QN12 and 2015QN04).

## Conflict of interest

The authors declare that the research was conducted in the absence of any commercial or financial relationships that could be construed as a potential conflict of interest.

## Publisher's note

All claims expressed in this article are solely those of the authors and do not necessarily represent those of their affiliated organizations, or those of the publisher, the editors and the reviewers. Any product that may be evaluated in this article, or claim that may be made by its manufacturer, is not guaranteed or endorsed by the publisher.
