# Supplementary material for: Key biomarkers and latent pathways of dysferlinopathy: Bioinformatics analysis and in vivo validation
Source: Front Neurol. 2022 Sep 20;13:998251. doi: 10.3389/fneur.2022.998251 (PMC9530905; doi:10.3389/fneur.2022.998251)
Supplement: Supplementary file 1 [file Table_1.DOCX]

Supplementary Table 1. Clinical features of 4 dysferlinopathy patients and 4 controls

|  | Age/Sex | Disease duration (years) | Symptoms of onset | CK levels (U/L) | Dysferlin  Immunostaining | EMG | *DYSF* sequencing | Biopsy site |
| --- | --- | --- | --- | --- | --- | --- | --- | --- |
| Dysferlinopathy group | | | | | | | | |
| 1 | 48/M | 7 | Limb weakness | 4545 | negative | myogenic damage | NM_003494, c.2810+1G>A  NM_003494, c.5525G>A | Quadriceps femoris |
| 2 | 33/M | 3 | Limb Weakness | 6803 | negative | myogenic damage | NM_003494, c.1284+1G>A  NM_003494, c.2875C>T | Biceps brachii |
| 3 | 27/M | 3 | Limb Weakness | 4531 | negative | myogenic damage | NM_003494, c.3112C>T  NM_003494, c.4228C>T | Tibialis anterior |
| 4 | 32/F | 2 | Limb Weakness | 9472 | negative | myogenic damage | NM_003494, c.5497G>T  NM_003494, c.4638+8C>G | Gastrocnemius |
| Control group | | | | | | | | |
| 5 | 19/M | 3 | Limb tremble | normal | normal | normal | - | Biceps brachii |
| 6 | 62/F | 0.5 | myalgia | normal | normal | normal | - | Gastrocnemius |
| 7 | 68/F | 0.5 | Fatigue, atrophy | normal | normal | normal | - | Quadriceps femoris |
| 8 | 43/F | 4 | myalgia | normal | normal | normal | - | Biceps brachii |
