# Supplementary material for: Key biomarkers and latent pathways of dysferlinopathy: Bioinformatics analysis and in vivo validation
Source: Front Neurol. 2022 Sep 20;13:998251. doi: 10.3389/fneur.2022.998251 (PMC9530905; doi:10.3389/fneur.2022.998251)
Supplement: Supplementary file 2 [file Table_2.docx]

Supplementary Table 2: Primer Sequences used for qRT-PCR.

|  | Forward | Reverse |
| --- | --- | --- |
| *GAPDH* | TGACTTCAACAGCGACACCCA | CACCCTGTTGCTGTAGCCAAA |
| *ERP29* | GGATCGCCAGGTGATTGAGAAG | TGGAAGGCAGTCAGGATGTTTAAGC |
| *GRN* | AGATGTCCCCTGTGATAATGTC | GATGTCTCTGGGGTGGGATAAG |
| *KPNA3* | CAGAAATCCACCGATTGATGAC | CCATACTGCTTGTTCACAAACA |
| *MVP* | CCCAACACTGCCCTCCATCTAAAG | ATCTCCACGACCTCCACTTCCTTC |
| *NFYB* | TGACAGTTCTACAACAGATGCT | GCTGTCCTCAGTATCATCATGA |
| *RNF128*  *PRKN* | AATGTGGCGTTTATGGATTAGC  GTGTGACAAGACTCAATGATCG | TGGTCACAAGCTTGGTAGTTAT  CCTGAAGTGATGGAGCTCTTTA |
