# Supplementary material for: Key biomarkers and latent pathways of dysferlinopathy: Bioinformatics analysis and in vivo validation
Source: Front Neurol. 2022 Sep 20;13:998251. doi: 10.3389/fneur.2022.998251 (PMC9530905; doi:10.3389/fneur.2022.998251)
Supplement: Supplementary file 3 [file Data_Sheet_1.PDF]

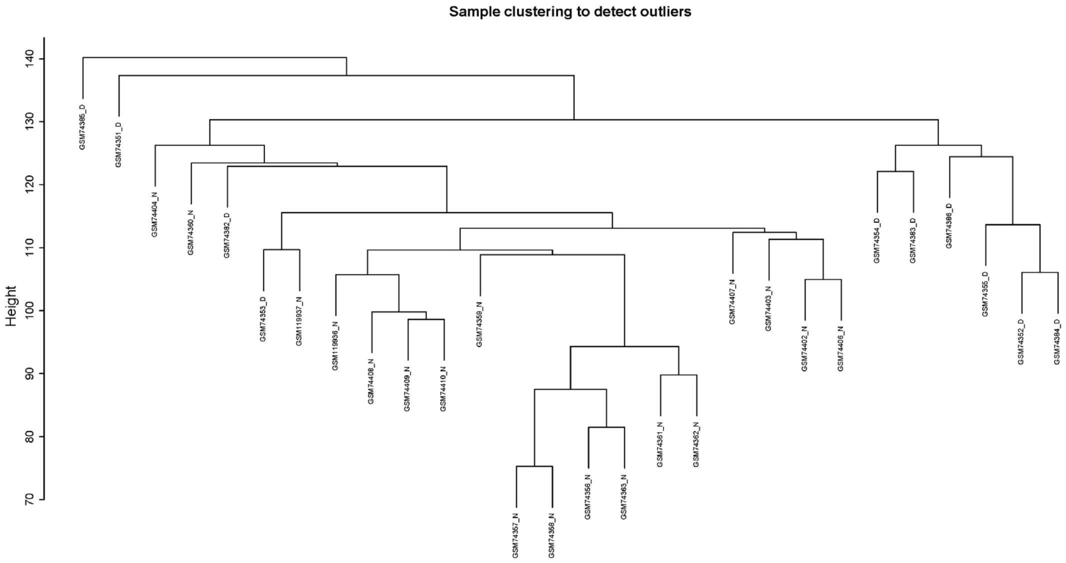

Supplementary Figure 1. Clustering dendrogram of samples. The abscissa represents the sample name while the ordinate represents the clustering height.
