# Supplementary material for: Key biomarkers and latent pathways of dysferlinopathy: Bioinformatics analysis and in vivo validation
Source: Front Neurol. 2022 Sep 20;13:998251. doi: 10.3389/fneur.2022.998251 (PMC9530905; doi:10.3389/fneur.2022.998251)
Supplement: Supplementary file 5 [file Data_Sheet_3.PDF]

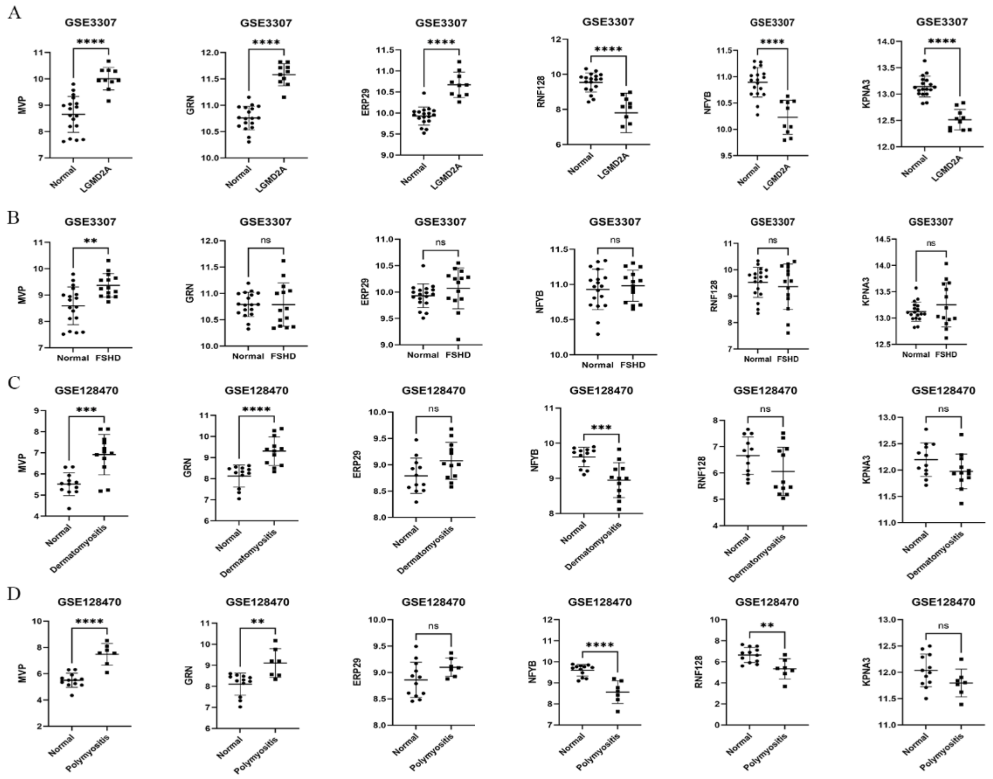

Supplementary Figure 3. Expression validation of six genes. (A) Expression of *MVP*, *GRN*, *ERP29*, *RNF128*, *NFYB*, *KPNA3* and *PRKN* in LGMD 2A (n=10) compared with controls (n=18) in the GSE3307 dataset. (B) Expression of *MVP*, *GRN*, *ERP29*, *RNF128*, *NFYB*, *KPNA3* and *PRKN* in FSHD (n=14) compared with controls (n=18) in the GSE3307 dataset. (C) Expression of *MVP*, *GRN*, *ERP29*, *RNF128*, *NFYB*, *KPNA3* and *PRKN* in dermatomyositis (n=12) compared with controls (n=12) in the GSE128470 dataset. (D) Expression of *MVP*, *GRN*, *ERP29*, *RNF128*, *NFYB*, *KPNA3* and *PRKN* in polymyositis (n=7) compared with controls (n=12) in the GSE128470 dataset. LGMD2A, Limb-girdle muscular dystrophy 2A; FSHD, facioscapulohumeral muscular dystrophy; *$p<0.05$; **$p<0.01$; ***$p<0.001$; ****$p<0.0001$; ns=non significance.
