# Supplementary material for: Key biomarkers and latent pathways of dysferlinopathy: Bioinformatics analysis and in vivo validation
Source: Front Neurol. 2022 Sep 20;13:998251. doi: 10.3389/fneur.2022.998251 (PMC9530905; doi:10.3389/fneur.2022.998251)
Supplement: Supplementary file 6 [file Data_Sheet_4.PDF]

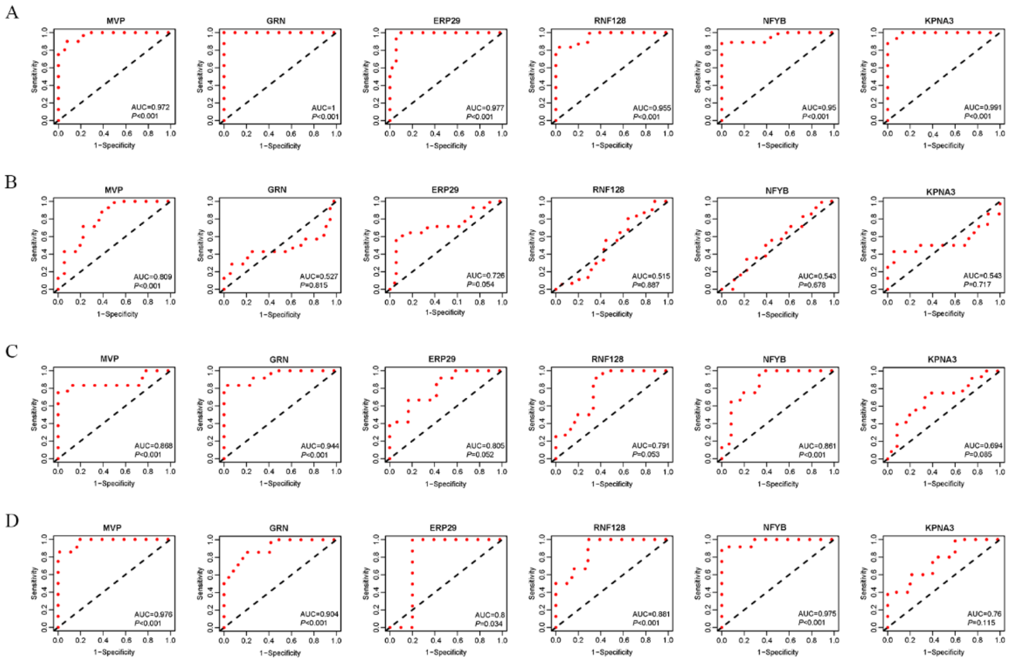

Supplementary Figure 4. Diagnostic efficiency of the six key genes. (A) The diagnostic value of six key genes in the LGMD 2A samples. (B) The diagnostic value of six key genes in the FSHD samples. (C) The diagnostic value of six key genes in the dermatomyositis samples. (D) The diagnostic value of six key genes in the polymyositis samples. LGMD2A, Limb-girdle muscular dystrophy 2A; FSHD, facioscapulohumeral muscular dystrophy.
