# Supplementary material for: Key biomarkers and latent pathways of dysferlinopathy: Bioinformatics analysis and in vivo validation
Source: Front Neurol. 2022 Sep 20;13:998251. doi: 10.3389/fneur.2022.998251 (PMC9530905; doi:10.3389/fneur.2022.998251)
Supplement: Supplementary file 4 [file Data_Sheet_2.PDF]

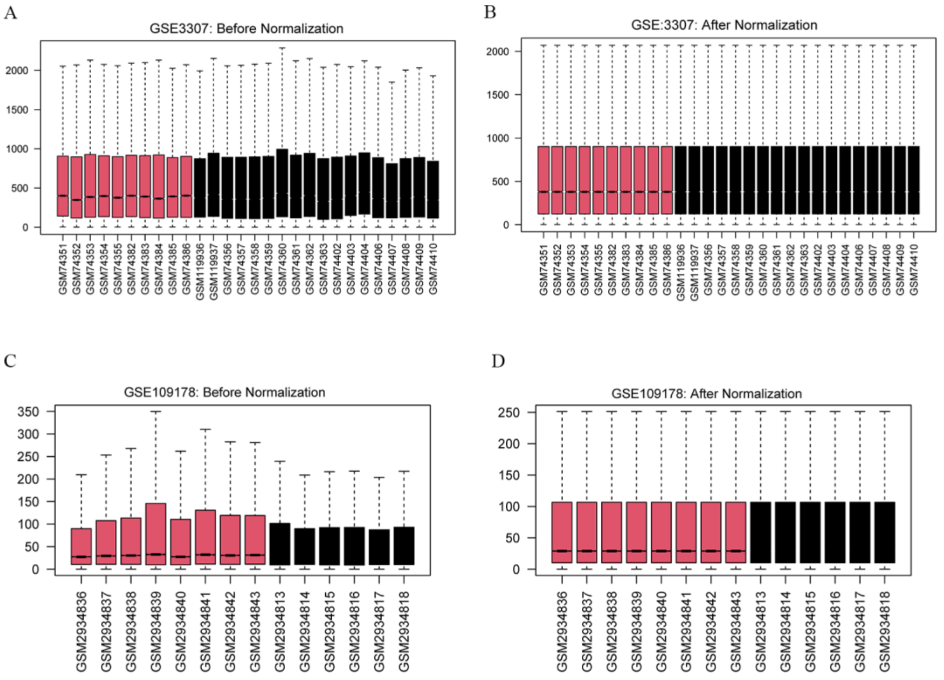

Supplementary Figure 2. Standardization of GSE3307 and GSE109178. (A-B) Gene expression data in GSE3307 before and after normalization. (C-D) Gene expression data in GSE109178 before and after normalization. The red bar represents dysferlinopathy samples, and the black bar represents normal controls.
